# Risk-Based Estimate of Human Fungal Disease Burden, China

**DOI:** 10.3201/eid2609.200016

**Published:** 2020-09

**Authors:** Ling-Hong Zhou, Ying-Kui Jiang, Ruo-Yu Li, Li-Ping Huang, Ching-Wan Yip, David W. Denning, Li-Ping Zhu

**Affiliations:** Huashan Hospital, Shanghai, China; and Fudan University, Shanghai (L.-H. Zhou, Y.-K. Jiang, L.-P. Huang, C.-W. Yip, L.-P. Zhu);; Peking University First Hospital, Beijing, China (R.-Y. Li);; Peking University, Beijing (R.-Y. Li);; National Clinical Research Center for Skin and Immune Diseases, Beijing (R.-Y. Li);; Wythenshawe Hospital, Manchester, UK (D.W. Denning);; University of Manchester, Manchester (D.W. Denning);; Global Action Fund for Fungal Infections, Geneva, Switzerland (D.W. Denning)

**Keywords:** fungal disease, burden, China, fungi, prevalence, risk factor

## Abstract

We conducted a systematic literature review to obtain risk population–based fungal disease incidence or prevalence data from China. Data were categorized by risk factors and extrapolated by using most recent demographic figures. A total of 71,316,101 cases (5.0% of the population) were attributed to 12 risk factors and 17 fungal diseases. Excluding recurrent *Candida* vaginitis (4,057/100,000 women) and onychomycosis (2,600/100,000 persons), aspergillosis (317/100,000 persons) was the most common problem; prevalence exceeded that in most other countries. Cryptococcal meningitis, an opportunistic infection, occurs in immunocompetent persons almost twice as often as AIDS. The pattern of fungal infections also varies geographically; *Talaromyces marneffei* is distributed mainly in the Pearl River Basin, and the Yangtze River bears the greatest histoplasmosis burden. New host populations, new endemic patterns, and high fungal burdens in China, which caused a huge impact on public health, underscore the urgent need for building diagnostic and therapeutic capacity.

Fungal diseases constitute a growing problem worldwide, causing a large, but poorly quantified, impact on public health (*1*). The incidence of fungal infections varies according to geographic region, socioeconomic conditions, and the number of persons with underlying conditions. China is one of the largest countries in the world (largest population and third largest land area). It has almost every type of weather niche, from the Pacific coast in the south to the snowy mountains in the Qinghai–Tibet Plateau, and even tropical rain forest. Many endemic fungal infections are present in China, along with globally distributed fungal pathogens. Although China has become the world’s second largest economy, it is still a developing country, with millions of impoverished citizens who are susceptible to fungal infections. Fungal keratitis, one of the major causes of avoidable blindness, has been relatively neglected (*2*). Moreover, old pathogens such as *Histoplasma* and *Talaromyces marneffei* (talaromycosis) have expanded (*3**,**4*); new hosts contributing to new therapies for malignant and autoimmune disease have increased (*5**,**6*); and new patterns, including aspergillosis in pulmonary tuberculosis (PTB) and chronic obstructive pulmonary disease (COPD), are emerging (*7**,**8*). In addition, the lack of effective drugs, shortages of well-trained medical care personnel, and unaffordable antifungal drugs result in severe outcomes. Therefore, an estimation of fungal disease burden is needed for China to increase public health awareness and facilitate effective interventions.

As in most other countries, fungal infections are not reportable in China, and the incidence and prevalence are difficult to calculate because of the lack of population-based surveillance data and few high quality epidemiology studies. The Chinese National Fungal Diseases Surveillance System (http://www.chifungi.cn) was established on May 18, 2019, but no data have been released yet. Even with this dearth of data, we have attempted to estimate the burden of fungal disease in China.

## Materials and Methods

### Study Procedures

**Figure 1:**
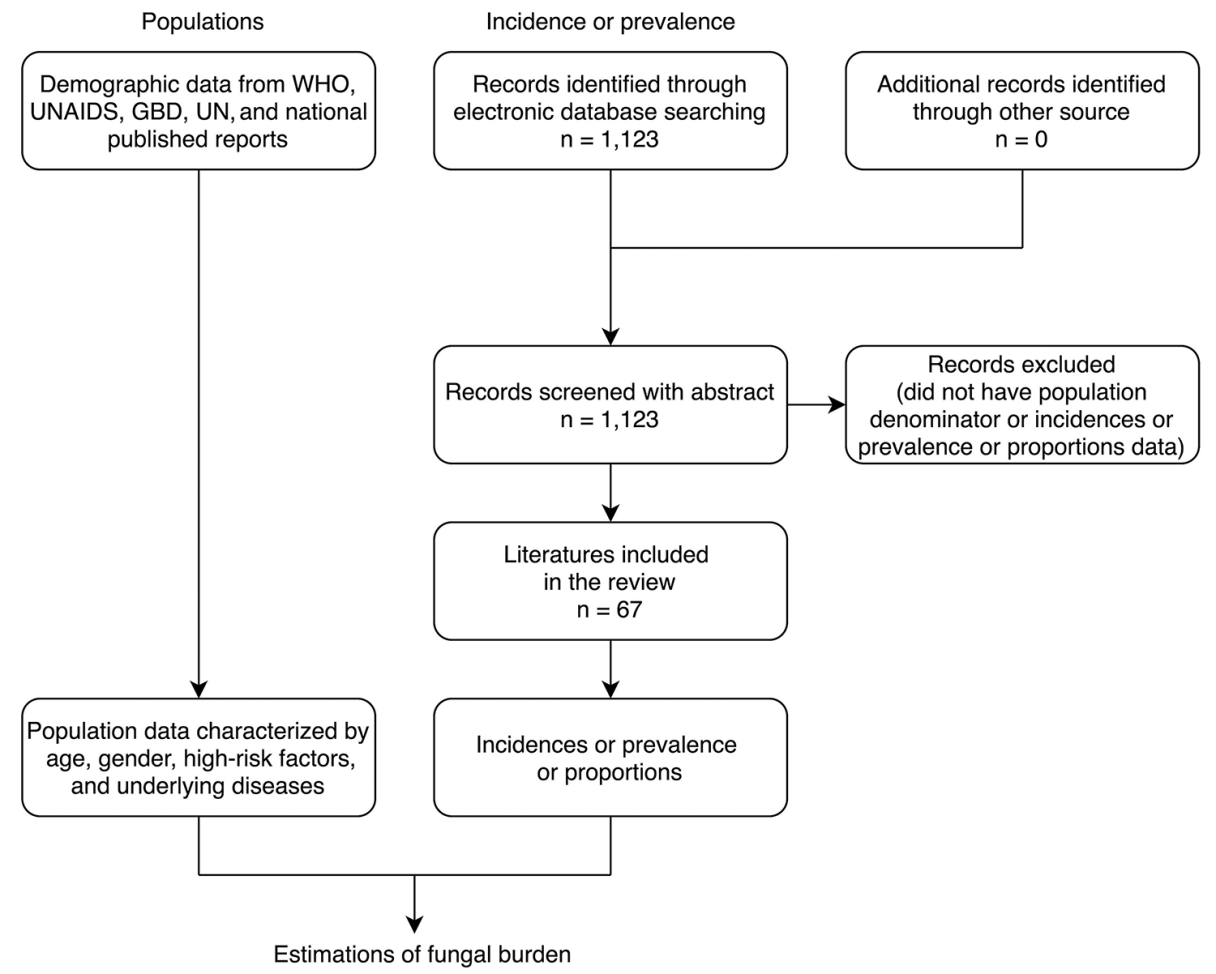
Flowchart of literature review for the human fungal disease burden in China. Reports published in English during January 1950–October 2019 were searched. GBD, Global Burden of Disease, Injuries, and Risk Factors Study; UNAIDS, the Joint Nations Program on HIV/AIDS; UN, United Nations Population Division; WHO, World Health Organization.

We conducted a literature review for published epidemiology papers that discussed fungal infections in China. If no epidemiological data existed for a particular fungal disease, we estimated the burden based on fungal infection incidence or prevalence and the specific populations at risk (Figure 1; Appendix Table 1).

### High-Risk Population Data

We obtained population statistics, including China’s total, child, and female populations 14–49 years of age, from the United Nations Population Division (*9*). We derived data on HIV/AIDS in China from the Joint Nations Program on HIV/AIDS (UNAIDS) (*10*); we used the same source to calculate the proportion of HIV patients on antiretroviral therapy (ART). We consulted the World Health Organization (WHO) tuberculosis report to obtain data on tuberculosis patients; we assumed that 5.6% of these patients died (*11*). The numbers of lung cancer and hematological malignancy cases were derived from Global Cancer Observatory (GLOBOCAN) reports (*5*); data related to transplant recipients were derived from China Organ Transplantation Registration System (COTR) (*12*). For other high-risk populations, we extracted data from relevant published reports (Table 1).

**Table 1:** Population characteristics in China, by age, gender, high-risk factors, and underlying diseases*

Population characteristic	No., in thousands	Reference
Total population	1,433,784	UN, 2019 (*9*)
Population of children 0–14 y	254,930	UN, 2020 (*9*)
Female population, 15–49 y	403,377	UN, 2020 (*9*)
Population >40 y	688,074	UN, 2020 (*9*)
People living with HIV	810	UNAIDS, 2017 (*10*)
Proportion of HIV patients on ART	40%	UNAIDS, 2017 (*10*)
Adults living with HIV and CD4 <200 cells/µL	106	Assumes a 5-y decline in immunity
AIDS related deaths	26	UNAIDS, 2017 (*10*)
Annual cases of TB	856	WHO 2017 (*11*)
Annual cases of pulmonary TB who survive	844	WHO 2017 (*11*)
Adults with asthma (4.2%) population	49,512	Huang, 2019 (*13*)
Adults with COPD (7.2% of population)	102,377	Zhu, 2018 (*14*)
Adults with COPD admitted to hospital each year (20.1%)	29,382	Zhu, 2018 (*14*)
Lung cancer	774.3	GLOBOCAN, 2018 (*1*)
Liver transplants per year	4.73	COTR (*12*)
Renal transplants per year	10.8	COTR (*12*)
Lung transplants per year	0.3	COTR (*12*)
Heart transplants per year	0.56	COTR (*12*)
Allogeneic stem cell transplants per year	5.0	Xu, 2016 (*15*)
Acute myelogenous leukemia	41.2	40% of GLOBOCAN leukemia and multiple myeloma total, 2018 (*1*)
No. patients on peritoneal dialysis	73.9	Wilkie and Davies, 2017 (*16*)
Intensive care unit beds	86.0	Murthy, 2012 (*17*)
Intensive care admissions surviving >24 h	5,126	Du, 2013 (*18*)

*ART, antiretroviral therapy; COPD, chronic obstructive pulmonary disease; COTR, China Organ Transplantation Registration System; TB, tuberculosis; UN, United Nations Population Division; UNAIDS, the Joint Nations Program on HIV/AIDS; WHO, World Health Organization.

### Selection of Studies for Incidence or Prevalence Data

We conducted a systematic literature review and identified published epidemiology papers. We searched Web of Science, PubMed, and Embase databases for all the eligible studies published during January 1, 1950–October 7, 2019. Studies selected for this analysis were published in English; we included population-based incidence studies, population-based surveillance systems, and national investigation data. If no available incidence or prevalence data from China were found, we considered published global or international data. All search strings are listed in Appendix Table 2 and studies contributing to estimates for each fungal disease are listed in Appendix Table 3. All the assumptions and calculations for different fungal diseases are detailed in Table 2.

**Table 2:** Assumptions and calculations for the estimations of fungal disease burden, China*

Fungal diseases†	Assumptions	Calculations
Candidemia	1. Candidemia episodes in ICU = (ICU beds × 365/median length of ICU stay) × (rate of candidemia in ICU/1,000 admissions)	Candidemia = Candidemia episodes in ICU/0.20
	2. 20% of candidemia episodes in Asia occur in ICU	
*Candida* peritonitis	Rate of *Candida* peritonitis is 50% of cases of candidemia in ICU	*Candida* peritonitis = candidemia in ICU × 50%
*Candida* peritonitis CAPD	1. 3.7% were *Candida* peritonitis in all episodes of infection	*Candida* peritonitis CAPD = peritoneal dialysis × 0.27 × 3.7%
	2. Overall infection incidence was 0.27 episodes/patient/ year	
Oral candidiasis	Assumed to occur in 45% of AIDS cases annually	Oral candidiasis = AIDS × 45%
Esophageal candidiasis	Assumed to occur in 20% of HIV patients not on ART and 5% of patients taking ART annually	Esophageal candidiasis = (0.2 × HIV patients not on ART) + (0.05 × HIV patients on ART)
RVVC	Assumed to occur in 7.2% of the female population 15–49 years of age	RVVC = (female population 15–49) × 7.2%
IA	1. In hematologic malignancy, annual incidence of all leukemias and multiple myeloma × 40% × 13%	IA = IA in hematologic malignancy + IA in solid and HSCT recipients + IA in lung cancer patients + IA in COPD patients + IA in HIV/AIDS patients
	a. Acute myeloid leukemia estimated at 40% of annual incidence of leukemias and multiple myeloma	
	b. 13% of patients with acute myeloid leukemia developed IA	
	2. IA in solid and HSCT recipients: assumed 10% in a-HSCT recipients, 2% of renal transplants, 6% of heart transplants, 4% of liver transplants, 20% of lung transplants	
	3. IA in 2.6% of patients with lung cancer	
	4. IA in COPD: COPD patients × 20.9% × 3.9%	
	a. Annual hospitalization rate for COPD = 20.9%	
	b. 3.9% of hospitalized COPD patients developed IA	
	5. IA in 4% of HIV/AIDS patients	
CPA	1. TB-related CPA: assuming rate of 22% among patients with lung cavities, 2% of patients without cavities	Total CPA = TB-related CPA × 3
	2. 22% of patients with pulmonary TB have residual lung cavities	
	3. One third of underlying diseases of CPA are TB	
ABPA	1. 4.2% of adults in China have asthma	ABPA = adults with asthma × 2.5%
	2. 2.5% of adults with asthma have ABPA	
SAFS	1. Assume a conservative 33% rate of fungal sensitization in patients with severe asthma	SAFS = adult population × 33% × 10%
	2. 10% of adults with asthma have severe asthma	
CM	1. 7.1% of patients with HIV/AIDS	CM = (7.1% × HIV/AIDS patients / 21%) + 0.43/100,000 × child population
	2. HIV-related CM is 21% of total CM	
	3. Annual incidence of 0.43/100,000 in children	
PCP	1. 22.4% of HIV-positive patients during a 2y period	PCP = 22.4% × HIV/AIDS patients / 2 / 70.22%
	2. HIV-related PCP is 70.22% of total PCP	
Talaromycosis	Assume 20% of AIDS patients geographically exposed, attack rate 15%	Talaromycosis = HIV/AIDS patients × 20% × 15%
Histoplasmosis	Assume 67% of AIDS patients geographically exposed, attack rate 5%	Histoplasmosis = HIV/AIDS patients × 5% × 67%
Mucormycosis	Assume prevalence is 0.2/100,000 in total population	Mucormycosis = total population × 0.2/100,000
Fungal keratitis	0.007% of total population	Fungal keratitis = total population × 0.007%
Onychomycosis	2.6% of total population	Onychomycosis = total population × 2.6%

*ABPA, allergic bronchopulmonary aspergillosis; ART, antiretroviral therapy; CAPD, continuous ambulatory peritoneal dialysis; CM, cryptococcal meningitis; CPA, chronic pulmonary aspergillosis; HSCT, hematopoietic stem cell transplant: IA, invasive aspergillosis; ICU, intensive care unit; PCP, pneumocystis pneumonia; RVVC, recurrent *Candida* vaginitis; SAFS, severe asthma with fungal sensitization. †Example for reading the table: burden of candidemia = candidemia episodes in ICU / 0.20 = (ICU beds × 365 / median length stay in ICU) × (rate of candidemia in ICU/1000 admissions) /0.20 = (86,027 × 365 / 6.126) × (3.2 / 1000) / 0.20 = 82,011.

## Analysis of Data

### Candidiasis Burden Estimations

We estimated burdens of invasive candidiasis, *Candida* peritonitis, *Candida* peritonitis as a complication of chronic ambulatory peritoneal dialysis (CAPD), and recurrent vulvovaginal candidiasis (RVVC). To estimate invasive candidiasis, we first assessed candidemia incidence in intensive care units (ICUs). Because »20% of candidemia episodes in Asia occur in ICUs (*19*), we used these data to estimate annual incidence for all units. We then estimated *Candida* peritonitis by assuming that there were 2 episodes of candidemia per episode of intraabdominal candidiasis in the ICU, based on a large prospective study (*20*). In addition, we estimated *Candida* peritonitis in CAPD using data from the First Affiliated Teaching Hospital in Tianjin (*21*). For RVVC, when prevalence data were not available, we used the rate of women with RVVC from a recent global estimate (*22*). We assumed that esophageal candidiasis occurred in 20% of patients with HIV who were not on ART and 5% of those taking ART annually (*23*). Oral candidiasis was estimated only in patients with HIV; we assumed that it occurs in 45% of patients with AIDS annually (*24*).

### Aspergillosis Burden Estimations

We calculated burdens of invasive aspergillosis (IA), chronic pulmonary aspergillosis (CPA), allergic bronchopulmonary aspergillosis (ABPA), and severe asthma with fungal sensitization (SAFS). We estimated the annual incidence of IA in hematological malignancy, solid and hematopoietic stem cell transplant (HSCT) recipients, lung cancer, COPD, and deaths from AIDS. We estimated that acute myeloid leukemia accounted for 40% of the annual incidence of all leukemias and multiple myeloma in 2018 (*5*). We took the rate of IA of 13% in hematological malignancy from a study from Taiwan (*25*), where mold-active prophylaxis was not given, and an equal number of cases were seen in all other leukemia and lymphoma cases. Among allogeneic HSCT recipients, we assumed an IA rate of 10% and rates in solid organ transplant recipients of 2% (renal), 6% (heart), 4% (liver), and 20% (lung) (*23*). For patients with lung cancer, we used a rate of 2.6% from a large study from China (*26*). To estimate the annual incidence of IA in patients with COPD, we used a recent study in Guangzhou Province, which found that the rate of IA in hospitalized patients with COPD was 3.9% (*27*). The annual hospitalization rate was a mean of 20.9%, and the number of patients with COPD came from a systematic review (*28*), from which we estimated the hospitalized patients with COPD. Although this information was not reported from China, we assumed a 4% autopsy incidence of IA in patients with AIDS (*29*).

We used the WHO 2017 figures for PTB to calculate CPA (*11*). We calculated CPA incidence after PTB based on our previous estimate, assuming that 22% of patients are left with a pulmonary cavity and that 22% of these patients develop CPA each year, as did 2% of those without a cavity (*30*). This calculation derives an annual incidence of CPA, which we converted to a 5-year period prevalence by assuming a 15% annual death or surgical resection rate. Given that PTB is one of several underlying causes of CPA, we conservatively assumed that PTB was primarily responsible for 33% of all CPA cases (*31*).

The reported rate of asthma in adults in China has increased from 1.42% in 2012 to 4.2% in 2019 (*13*). Ma et al. ascertained that 2.5% of these patients had ABPA (in secondary care) (*32*). Severe asthma proportion of adult asthmatics was estimated at 10%, as in other country estimates (*23*). We used a conservative fungal sensitization rate of 33% (as in other countries) to estimate the number of SAFS (*23*). No estimation was made about cystic fibrosis in China because few patients currently survive to adulthood.

### HIV-Related Infection Burden Estimation

We estimated burdens of cryptococcal meningitis (CM), *Pneumocystis* pneumonia (PCP), talaromycosis, and histoplasmosis. We ignored other HIV-related infections because of the lack of population-based data. We derived data for patients with AIDS from those who had a 5-year decline in CD4 counts to <200 cells/mL in the total population of HIV patients. We estimated the annual incidence of CM at 8% in patients with AIDS (CD4 count <200 cells/mL) (*33*) and estimated the overall CM annual incidence based on the assumption that the proportion of HIV-positive patients with CM was 21% (*34*). In children, 23 cases of CM were diagnosed over a 5-year period (2007–2012) in Shijiazhuang, giving an annual incidence of 0.43/100,000 (*35*). We conservatively estimated the 2-year incidence of PCP at 22% of patients with AIDS (*36*), which comprises 70% of total cases (*37*). Only 20% of the HIV population was assumed to be geographically at risk for infection with *T. marneffei*; the attack rate was 15% in patients with AIDS (*3*). Disseminated histoplasmosis was assumed to occur in 5% of the geographically exposed population (estimated at 67%) of patients with AIDS (*4*).

### Mucormycosis, Fungal Keratitis, and Onychomycosis Burden Estimation

Mucormycosis is a rare fungal infection; the prevalence rate is 0.2–140.0/1 million population (*38*). We used a global prevalence rate (2.0/1 million population) to estimate the burden. To estimate the burden of fungal keratitis, which is usually caused by *Fusarium* spp. and *Aspergillus* spp. in China, we used the overall prevalence of 0.007% according to a multicenter study (*2*). We used the global prevalence rate (2.6%) from 11 population-based studies to estimate the onychomycosis burden (*39*).

### Epidemiology Maps

For talaromycosis and histoplasmosis, which showed new endemic trends, we prepared epidemiology maps according to the number of reported cases in China. We searched the PubMed database for articles published in China during January 1, 1950–October 7, 2019. Reports published in English were included. Search strings and references contributing to the talaromycosis map are listed in Appendix Table 4, and those contributing to the histoplasmosis map are listed in Appendix Table 5.

### Prediction of HIV-Related Invasive Fungal Burden

We made a simple prediction model to estimate the HIV-related fungal burden by 2050. We derived the prediction data for total population in the next 50 years from UN data (*40*), and we collected data on HIV and AIDS cases during 2012–2017 to make a linear regression model to predict the number of HIV cases in 2050. If early testing and ART are at the current level, we estimate that 20.4% of HIV patients will develop advanced HIV disease over time (*10*). Based on our assumptions, we also predicted the burdens of invasive fungal diseases, including CM, PCP, and talaromycosis.

## Results

### Population Profiles

According to the UN data, the population of China was »1.4 billion in 2019, of whom 18% were children; 688 million adults were >40 years of age, of whom 403 million were women 15–49 years of age (*9*). The current total number of reported HIV infections in China is 810,000, and there were 26,000 AIDS-related deaths in 2017. Thus, 784,000 persons were living with HIV in China in 2019, of whom 60% were not receiving ART and 165,018 had AIDS (CD4 <200) (*10*). The detailed population characteristics and high-risk populations are described in Table 1.

### Candidiasis Burden

The overall fungal burden in China, according to major risk factors, is summarized in Table 3. In 2012, there were an estimated 86,027 intensive care beds, in China and the rate of candidemia in ICU was documented at 3.2/1000 ICU admissions. The median length of stay in the ICU in China is 6.1 days. Thus, there are 16,402 candidemia episodes in the ICU, and we estimated a total of 82,011 episodes of candidemia per year in all units. Although *C. albicans* remains the most common species associated with candidiasis in ICU patients, other non–*albicans Candida* (NAC) is becoming increasingly common, and patients with NAC usually have longer antifungal therapy, longer ICU or hospital stay, and slightly higher death rates (*41*).

**Table 3:** Summary of fungal infection burden in China according to major risk factors*

Infection	No. infections per underlying disorder per year					Total no. cases	Rate/100,000 population
	None	HIV/AIDS	Respiratory	Cancer	ICU		
Candidemia	NE	NE	NE	65,609	16,402	82,011	5.72
*Candida* peritonitis							
ICU + surgery	NE	NE	NE	NE	8,939	8,939	0.62
CAPD	738	NE	NE	NE	NE	738	0.05
Oral candidiasis	NE	74,258	NE	NE	NE	74,258	5.18
Esophageal candidiasis	NE	49,204	NE	NE	NE	49,204	3.43
Recurrent *Candida* vaginitis	29,082,000	NE	NE	NE	NE	29,082,000	4,056.68†
IA	NE	1,040	1,145,908	31,800	NE	1,178,748	82.21
CPA	NE	NE	488,716	NE	NE	488,716	34.09
ABPA	NE	NE	1,237,797	NE	NE	1,237,797	86.33
SAFS	NE	NE	1,633,892	NE	NE	1,633,892	113.96
CM	26,249	13,086	NE	26,172	NE	65,607	4.57
PCP	NE	18,482	NE	9,241	NE	27,723	1.93
Talaromycosis	NE	4,951	NE	NE	NE	4,951	0.35
Mucormycosis	2,868	NE	NE	NE	NE	2,868	0.20
Fungal keratitis	100,365	NE	NE	NE	NE	100,365	7.00
Onychomycosis	37,278,384	NE	NE	NE	NE	37,278,384	2,600.00
Total burden	66,490,604	161,021	4,506,313	132,822	25,341	71,316,101	7,002.32

*ABPA, allergic bronchopulmonary aspergillosis; ART, antiretroviral therapy; CAPD, continuous ambulatory peritoneal dialysis; CM, cryptococcal meningitis; CPA, chronic pulmonary aspergillosis; IA, invasive aspergillosis; ICU, intensive care unit; NE, no estimation could be made because of the lack of data; PCP, pneumocystis pneumonia; SAFS, severe asthma with fungal sensitization.  †Female population only.

We also estimated 8,201 cases of postsurgical *Candida* peritonitis (intraabdominal candidiasis) by making the assumption that the rate of *Candida* peritonitis is 50% of cases of candidemia in the ICU. Given that there were 73,871 patients on CAPD in China in 2017, we estimated 738 peritonitis cases by using the *Candida* peritonitis episode rate of 0.01/patient-year.

**Figure 2:**
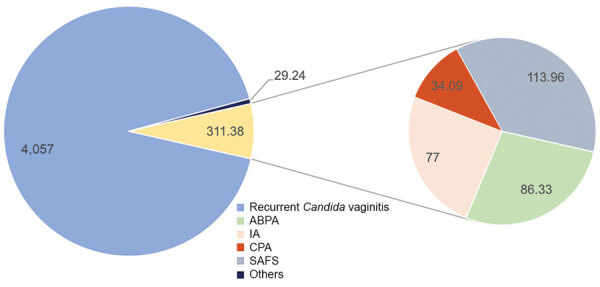
Estimated annual incidence (cases/100,000 population) of common fungal diseases in China. ABPA, allergic bronchopulmonary aspergillosis; CPA, chronic pulmonary aspergillosis; IA, invasive aspergillosis; SAFS, severe asthma with fungal sensitization.

Except for cutaneous disease, recurrent *Candida* vaginitis is the most common fungal disease, with aspergillosis, including IA, CPA, ABPA, and SAFS, next (Figure 2). We used the base case of RVVC prevalence in adult premenopausal women (15–49 years of age) previously published: 29,082,000 (range 21,812,000–36,353,000) affected women (*22*). In addition, 74,258 cases of oral candidiasis and 49,240 cases of esophageal candidiasis were expected annually in patients with HIV.

### Aspergillosis Burden

We obtained an estimate of 1,178,747 cases of IA (82.1/100,000 population). We estimated 32,840 cases in immunocompromised patients and those with cancer; of these, 20,000 were in patients with lung cancer and 1,040 in patients with AIDS. The remainder of IA cases in this immunocompromised group were in patients with hematologic malignancies, lymphoma, and transplants. We also calculated 1,145,908 IA cases derived from COPD.

In China, there were 844,500 survivors of tuberculosis in 2017 (*11*). We expect an annual incidence of 51,683 cases of tuberculosis-related CPA, and we estimated a 5-year period prevalence of 162,905 cases. Because tuberculosis probably comprises only one third of underlying CPA, the total period prevalence estimate is 488,716 cases (34/100,000 population).

Nearly 50 million adults with asthma live in China; of these, 10% have severe asthma. Regarding ABPA, the assumption is that 2.5% of adult asthmatics are affected, leading to a prevalence of 1,237,797 cases. Among patients with severe asthma, we estimate that 1.6 million have SAFS.

### Burdens of HIV-Related Infections

CM occurs mainly in immunocompromised populations other than HIV patients in China, as well as in immunocompetent individuals. We thus obtained an estimate of adult CM: 13,086 in patients with AIDS and 26,172 each in immunocompromised and in immunocompetent persons. For CM in children, we estimated 77 cases annually, with an annual incidence of 0.43/100,000 population. We calculated a total of 65,507 CM cases.

We estimated the number of patients with PCP in China as 27,723 (18,482 with HIV and 9,241 with other immunocompromised conditions). This estimate implies an annual incidence of 1.93/100,000 population.

**Figure 3:**
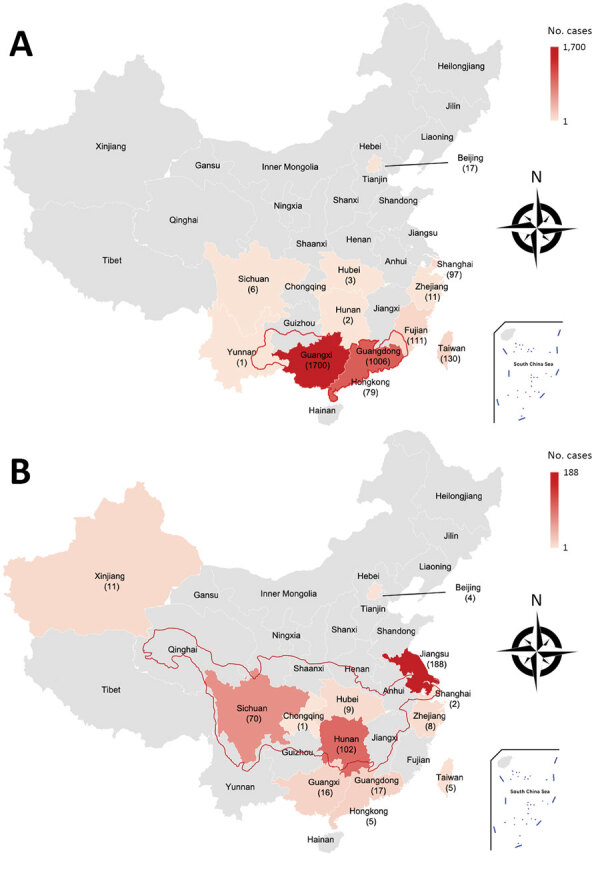
Epidemiology maps for talaromycosis and histoplasmosis, according to the number of reported cases, China. A) Map for talaromycosis. Red border indicates Pearl River Basin. B) Map for histoplasmosis. Red border indicates Yangtze River region. Reports published in English during January 1950–October 2019 were searched.

We estimated 4,951 talaromycosis cases in patients with AIDS in southern China. From the literature review, we identified 3,163 cases from 12 different provinces. The provinces with the highest prevalence are Guangxi and Guangdong, which each reported >1,000 cases (Figure 3, panel A). Both provinces are located in Pearl River Basin, possibly indicating an endemic trend.

**Figure 4:**
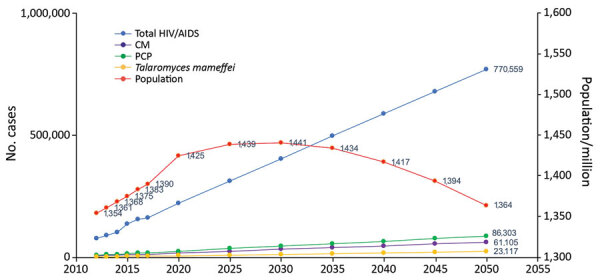
Prediction of HIV-related invasive fungal burden in China by 2050, based on ART and HIV-related disease incidence levels for 2012–2017. ART, antiretroviral therapy; CM, cryptococcal meningitis; PCP, pneumocystis pneumonia.

We identified 380 histoplasmosis cases in China from the literature review (Figure 3, panel B). Most of the cases were reported in the region where the Yangtze River flows, also suggesting a new endemic pattern in China. Disseminated histoplasmosis in patients with AIDS was assumed to affect 5,528 persons annually, but we were unable to estimate the burden in non–HIV-infected persons or the burden of chronic pulmonary histoplasmosis. For the prediction of HIV-related invasive fungal burden by 2050, using annual data from 2012–2017 and extrapolating with our estimates, we expect 86,303 cases of PCP, 61,105 cases of CM, and 23,117 cases of *T. marneffei* infection (Figure 4).

### Mucormycosis, Fungal Keratitis, and Onychomycosis Burden

We estimated mucormycosis using the global prevalence rate and calculated 2,868 cases. We estimated fungal keratitis based on the total population and estimated 100,365 cases of fungal keratitis annually in China. We estimated 37,278,384 cases of onychomycosis using the global data.

## Discussion

In this study, we estimated that 71 million persons suffer from a fungal disease in China. A total of 2.4% of the population is affected (excluding onychomycosis because it is superficial), similar to results in other reports from Senegal, Brazil, France, Korea and Germany; the prevalence range is 1.6%–3.6% (*1*). Multiple new host risk factors other than HIV/AIDS or hematologic malignancy, especially COPD, asthma, and lung cancer, are associated with fungal disease. We found that even immunocompetent children and women may develop fungal diseases. Chronic respiratory diseases, notably PTB and COPD, are risk factors for all manifestations of aspergillosis. Old pathogens, including *T. marneffei* and *Histoplasma capsulatum*, exhibit new endemic trends; the Pearl River Basin bears the greatest burden for *T. marneffei* and the Yangtze River for *H. capsulatum*. Our study contributes to the currently limited data on the burden of fungal disease in China and provides a basis for public health and research priorities.

The incidence of candidiasis has increased in recent years (*17*). Candidemia is probably underestimated, as we have used only ICU data to explore the total burden of all high-risk populations. Considering the wide use of broad-spectrum antimicrobial drugs and the demographic shift with a largely increasing elderly population, we expect infections to be on the rise.

We have estimated the oral and esophageal candidiasis burdens in HIV-positive patients; these are certainly underestimates of these infections, because many of the populations at risk could not be assessed, such as patients with cancer, those taking oral or inhaled corticosteroids, and newborns. Although the proportions of oral or esophageal candidiasis in this kind of populations might be small, given the relatively large size compared to the rather small HIV population, these cases could multiply our estimates. In addition, oral and esophageal candidiasis and colonization are associated with mucosal malignancy and particularly associated with high alcohol consumption (*42*). If the high number of unsuspected cases of esophageal candidiasis based on data from South Korea is also true in China, this association could contribute to the high number of esophageal cancers seen annually in China (307,359 cases) (*43*), which could further increase the social and economic burden.

IA is usually severe and fatal, unless diagnosed early. Although profoundly immunocompromised patients are at higher risk, the enormous estimate for China is mostly driven by COPD (97%). Our estimate of IA prevalence in liver transplant recipients of 4% is higher than the report from China at 1.7% (*44*), but that study was based on histology or culture alone, which is much less sensitive than *Aspergillus* antigen detection. The same applies to renal transplant recipients (*45*). Other underlying conditions were newly recognized risk factors for IA, such as diabetes mellitus, systemic lupus erythematosus, and postoperative and burn infections related to contaminated air in hospitals; these were not included in our estimation because of the unavailable incidence rate. Nevertheless, the number of IA cases could also be overestimated because *Aspergilli* are common fungi in the environment, and a positive result from non–aseptic fluid culture does not always represent disease. On the other hand, we have not estimated IA in most medical ICU patients, and not included the potential for IA complicating annual influenza cases or an epidemic.

In contrast ot other countries, where CM is often diagnosed in patients with HIV or immunocompromised patients, in China, a high proportion of cryptococcosis was reported in immunocompetent persons (*34*). Jiang et al. reported on 159 HIV-negative patients with CM, of whom 85 were normal hosts; however, whether these persons were immunocompetent is unknown, because several genetic predisposition factors for CM have been found in the ethnic Chinese population (*46*). Most large-scale studies have been conducted in adults; few were dedicated to pediatric populations. Although children account for only 0.9%–2.0% of all cryptococcal cases, the death rate is high, up to 43% (*47*). In a 12-year retrospective study in Beijing, 53 pediatric case-patients were encountered, of whom 41 had no underlying conditions (*48*). However, the denominator was unavailable. Only an annual incidence of 0.43/100,000 HIV-negative children was reported from the Acute Meningitis-Encephalitis Syndrome Surveillance project (*35*). Because of the lack of surveillance networks in China, additional studies are required, especially for immunocompetent patients.

Although histoplasmosis is a common endemic mycosis in North America, several sporadic cases were reported in China, especially in the Yangtze River region, which was traditionally thought to be nonendemic for *Histoplasma capsulatum* (*3*). However, >300 histoplasmosis cases have been reported since 1990, and only 17 were identified as imported cases, indicating many authochthonous cases in China (*3*). *T. marneffei* infection, the other fatal endemic opportunistic fungal infection disease in Asia, was reported mostly in the southern part of China, possibly linked to an altered microeukaryotic community in subtropical rivers caused by global warming (*49*). Because this disease was associated mainly with HIV/AIDS, we estimated the incidence only in HIV-positive patients. In addition, given the low national reported statistics of HIV infection (*3*), the estimation of talaromycosis burden may be far underestimated. More large population-based studies are needed to better clarify the frequency of these fungal infections in these at-risk patients.

The government of China has worked to improve healthcare over the past 2 decades. However, HIV remains a major public health issue, showing the fastest growth among 45 infectious diseases during 2004–2013 with an annual percentage change of 16.3% (*50*). Therefore, we made a prediction of HIV-related opportunistic fungal infections. According to our data, it is likely that CM, PCP, and *T. marneffei* infection are major health burdens, which call for much more clinical training, financial support, and public policies.

Even though the incidence rate was low compared with those for bacterial and viral infections, our study represents a heavy fungal burden considering the immense population base and high mortality of nonsuperficial mycoses. The drawbacks of our study are the few studies conducted in the country for some infections; the prevalence or incidence are not available for those diseases, including sporotrichosis and some dermatophytosis. Epidemiologic studies are required and population-based surveillance data remain to be estimated, both nationally and regionally. Improved epidemiologic data are necessary for better awareness, better diagnostics, and better therapies.

Appendix Additional information on the study of human fungal disease burden, China.
