# Supplementary material for: Risk-Based Estimate of Human Fungal Disease Burden, China
Source: Emerg Infect Dis. 2020 Sep;26(9):2137–47. doi: 10.3201/eid2609.200016 (PMC7454105; doi:10.3201/eid2609.200016)
Supplement: Appendix — Additional information on the study of human fungal disease burden, China. [file 20-0016-Techapp-s1.pdf]

Article DOI: https://doi.org/10.3201/eid2609.200016

Risk-Based Estimate of Human Fungal Disease Burden, China

Appendix

Appendix Table 1. Definitions of fungal diseases*

Fungal disease	Definition
Candidiasis	
Candidemia	Presence in the blood of fungi of the genus *Candida*
Candida peritonitis	Intraabdominal infection with *Candida* spp., often with coexisting bacterial infection, involving the peritoneum in most cases
Candida peritonitis (CAPD)	*Candida* peritonitis occurring during continuous ambulatory peritoneal dialysis
Oral candidiasis	Oral mucosa infection caused by *Candida* spp.
Esophageal candidiasis	Esophageal mucosa infection caused by *Candida* spp.
Recurrent *Candida* vaginitis (RVVC)	Four or more episodes of symptomatic vulvovaginal candidiasis within 1 year, at least some of which are mycologically confirmed
Aspergillosis	
IA	A rapidly progressive, often fatal infection caused by *Aspergillus* spp that occurs in patients who are immunosuppressed (e.g., leukemia) or critically ill (e.g., severe influenza)
CPA	A long-term *Aspergillus* infection (>3 months) of the lung; includes several disease manifestations, including aspergilloma, *Aspergillus* nodules, chronic cavitary pulmonary aspergillosis, and chronic fibrosing pulmonary aspergillosis
ABPA	A hypersensitivity reaction to *Aspergillus* species (generally *A. fumigatus*) that occurs almost exclusively in patients with asthma or, less commonly, cystic fibrosis
SAFS	Asthma with refractory symptoms, sensitization to $\geq$1 common fungi, but lacking diagnostic criteria for ABPA (such as *Aspergillus*-specific IgG)
HIV-related fungal diseases	
CM	A type of meningitis caused by *Cryptococcus*
PCP	A serious infection caused by the fungus *Pneumocystis jirovecii*, in both HIV-infected and other immunocompromised patients
Endemic fungal diseases	
Talaromyces marneffei infection	Talaromycosis is an invasive fungal infection, usually found in HIV-infected persons, caused by the dimorphic fungus *Talaromyces marneffei* (formerly *Penicillium marneffei*), which is endemic in Southeast Asia (in northern Thailand, Vietnam, and Myanmar), East Asia (in southern China, Hong Kong, and Taiwan), and South Asia (in northeastern India)

Fungal disease	Definition
Histoplasmosis	Histoplasmosis is caused by a fungus called *Histoplasma*. This fungus grows mainly in the central, southeastern, and mid-Atlantic United States and South Asia. The AIDS pandemic has elucidated a worldwide risk for histoplasmosis, including areas previously unknown to be endemic. In this article, only disseminated histoplasmosis is estimated.
Mucormycosis	A serious but rare fungal infection caused by a group of molds called Mucormycetes. Mucormycosis affects mainly diabetic or immunocompromised patients, occasionally others after trauma or intravenous drug abuse
Fungal keratitis	An infection of the cornea caused by a fungus
Onychomycosis	Fungal infection of the toenails or fingernails that may involve any component of the nail unit, including the matrix, bed, or plate, caused by many different fungi, including dermatophytes

* ABPA, allergic bronchopulmonary aspergillosis; CAPD, continuous ambulatory peritoneal dialysis; CM, cryptococcal meningitis; CPA, chronic pulmonary aspergillosis; IA, invasive aspergillosis; PCP, pneumocystis pneumonia; SAFS, severe asthma with fungal sensitization. Global prevalence was used to estimate RVVC, mucormycosis, and onychomycosis cases. When the Chinese or global prevalence or incidence were not available, burdens of some fungal diseases were not estimated, including endemic fungal infections like sporotrichosis and coccidioidomycosis and some dermatophytosis (tinea corporis, tinea capitis, tinea pedis, tinea cruris).

Appendix Table 2. Literature search results for fungal diseases*

Fungal disease	Search string	No. studies identified
Candidemia	(candidemia OR candidaemia) AND (incidence OR prevalence) AND China	60
Candida peritonitis	(*candida* peritonitis) AND (incidence OR prevalence) AND China	10
Candida peritonitis (CAPD)	(continuous ambulatory peritoneal dialysis OR CAPD) AND (*candida* peritonitis) AND (incidence OR prevalence) AND China	5
Oral candidiasis	(oral candidiasis) AND (incidence OR prevalence) AND China	76
Esophageal candidiasis	(oesophageal candidiasis OR esophageal candidiasis) AND (incidence OR prevalence) AND China	7
Recurrent *Candida* vaginitis	(recurrent candida vaginitis OR RVVC) AND (incidence OR prevalence) AND China	6
IA	(invasive aspergillosis) AND (incidence OR prevalence) AND China	89
CPA	(chronic pulmonary aspergillosis OR CPA) AND (incidence OR prevalence) AND China	79
ABPA	(allergic bronchopulmonary aspergillosis OR ABPA) AND (incidence OR prevalence) AND China	11
SAFS	(asthma with fungal sensitization OR SAFS) AND (incidence OR prevalence) AND China	10
CM	(cryptococcal meningitis) AND (incidence OR prevalence) AND China	47
PCP	(pneumocystis pneumonia OR pneumocystis jirovecii pneumonia OR PCP OR PJP) AND (incidence OR prevalence) AND China)	110
Talaromyces marneffei infection	(*Talaromyces marneffei* OR *Ienicillium marneffei* OR talaromycosis) AND (incidence OR prevalence) AND China	59
Histoplasmosis	(*histoplasma capsulatum* OR histoplasmosis) AND (incidence OR prevalence) AND China	23
Mucormycosis	(mucormycosis OR zygomycosis OR *mucorales*) AND (incidence OR prevalence); filters: meta-analysis, review, systematic reviews	270
Fungal keratitis	(fungal keratitis) AND (incidence OR prevalence) AND China	64
Onychomycosis	Onychomycosis AND (incidence OR prevalence); filters: meta-analysis, review, systematic reviews	197

*Reports published in English during January 1950–October 2019 were searched. ABPA, allergic bronchopulmonary aspergillosis; CAPD, continuous ambulatory peritoneal dialysis; CM, cryptococcal meningitis; CPA, chronic pulmonary aspergillosis; IA, invasive aspergillosis; PCP, pneumocystis pneumonia; PJP, pneumocystis jirovecii pneumonia; RVVC, recurrent *Candida* vaginitis; SAFS, severe asthma with fungal sensitization.

Appendix Table 3. Studies contributing to estimates of incidence or prevalence of fungal diseases in China*

Invasive aspergillosis
GLOBOCAN 2018: counting the toll of cancer. The Lancet. 2018;392:985.
Chen CY, Sheng WH, Tien FM, Lee PC, Huang SY, Tang JL, et al. Clinical characteristics and treatment outcomes of pulmonary invasive fungal infection among adult patients with hematological malignancy in a medical centre in Taiwan, 2008–2013. J. Microbiol Immunol Infect. 2018. (In press)
Lortholary O, Gangneux J-P, Sitbon K, Lebeau B, de Monbrison F, Le Strat Y, et al. Epidemiological trends in invasive aspergillosis in France: the SAIF network (2005–2007). Clin Microbiol Infect. 2011;17:1882–9.
Perkhofer S, Lass-Flörl C, Hell M, Russ G, Krause R, Hönigl M, et al. The Nationwide Austrian *Aspergillus* Registry: a prospective data collection on epidemiology, therapy and outcome of invasive mould infections in immunocompromised and/or immunosuppressed patients. Int J Antimicrob Agents. 2010;36:531–6.
Li L, Wang J, Zhang W, Yang J, Chen L, Lv S. Risk factors for invasive mold infections following allogeneic hematopoietic stem cell transplantation: a single center study of 190 recipients. Scand J Infect Dis. 2012;44:100–7.
Yan X, Li M, Jiang M, Zou L, Luo F, Jiang Y. Clinical characteristics of 45 patients with invasive pulmonary aspergillosis: retrospective analysis of 1711 lung cancer cases. Cancer. 2009;115:5018–25.
Zhu B, Wang Y, Ming J, Chen W, Zhang L. Disease burden of COPD in China: a systematic review. Int J Chron Obstruct Pulmon Dis. 2018;13:1353–64.
Denning DW, Pleuvry A, Cole DC. Global burden of chronic pulmonary aspergillosis as a sequel to tuberculosis. Bull WHO. 2011;89:864–72.
Fang L, Gao P, Bao H, Tang X, Wang B, Feng Y, et al. Chronic obstructive pulmonary disease in China: a nationwide prevalence study. Lancet Respir Med. 2018;6:421–30.
Xu H, Li L, Huang WJ, Wang LX, Li WF, Yuan WF. Invasive pulmonary aspergillosis in patients with chronic obstructive pulmonary disease: a case control study from China. Clin Microbiol and Infect. 2012;18:403–8.
Denning DW. Minimizing fungal disease deaths will allow the UNAIDS target of reducing annual AIDS deaths below 500 000 by 2020 to be realized. Phil Trans Roy Soc B. 2016;371:20150468.
Chen J, Yang Q, Huang J, Li L. Risk factors for invasive pulmonary aspergillosis and hospital mortality in acute-on-chronic liver failure patients: a retrospective-cohort study. Int J Med Sci. 2013;10:1625–31.
Mokdad AA, Lopez AD, Shahraz S, Lozano R, Mokdad AH, Stanaway J, et al. Liver cirrhosis mortality in 187 countries between 1980 and 2010: a systematic analysis. BMC Med. 2014;12:145.
Zhang X, Yang M, Hu J, Zhao H, Li L. Epidemiology of invasive pulmonary aspergillosis in patients with liver failure: Clinical presentation, risk factors, and outcomes. J Int Med Res. 2017;46:819–27.
**Chronic pulmonary aspergillosis**
World Health Organization. Tuberculosis country proviles [cited 2019 Oct 7]. http://www.who.int/tb/country/data/profiles/en/index.html
Smith NL, Denning DW. Underlying conditions in chronic pulmonary aspergillosis including simple aspergilloma. Eur Respir J. 2010;37:865–72.
Denning DW, Pleuvry A, Cole D. Global burden of chronic pulmonary aspergillosis as a sequel to pulmonary tuberculosis. Bull World Health Organ. 2011;89:864–72.

Invasive aspergillosis
He B, Wan C, Zhou W, Rui Y, Shi Y, Su X. Clinical profile and surgical outcome for different types of chronic pulmonary aspergillosis. Am J Transl Res. 2019;11:3671–79.
Zhan M, Xu B, Zhao L, Li B, Xu L, Sun Q, et al. The serum level of IL-1B correlates with the activity of chronic pulmonary aspergillosis. Can Respir J. 2018;2018:8740491.
Li H, Rui Y, Zhou W, Liu L, He B, Shi Y, et al. Role of the aspergillus-specific IgG and IgM test in the diagnosis and follow-up of chronic pulmonary aspergillosis. Front Microbiol. 2019;10:1438.
Yao Y, Zhou H, Shen Y, Yang Q, Ye J, Fu Y, et al. Evaluation of a quantitative serum *Aspergillus fumigatus*-specific IgM assay for diagnosis of chronic pulmonary aspergillosis. Clin Respir J. 2018;12:2566–72.
Ma X, Wang K, Zhao X, Liu Y, Yu X, Li C et al. Prospective study of the serum *Aspergillus*-specific IgG, IgA and IgM assays for chronic pulmonary aspergillosis diagnosis. BMC Infect Dis. 2019;19:694.
Lee JJ, Chong PY, Lin CB, Hsu AH, Lee CC. High resolution chest CT in patients with pulmonary tuberculosis: characteristic findings before and after antituberculous therapy. Eur J Radiol. 2008;67:100–4.
Smith N, Denning DW. Underlying pulmonary disease frequency in patients with chronic pulmonary aspergillosis. Eur Resp J. 2011;37:865–72.
Allergic bronchopulmonary aspergillosis
Denning DW, Pleuvry A, Cole DC. Global burden of allergic bronchopulmonary aspergillosis with asthma and its complication chronic pulmonary aspergillosis in adults. Med Mycol. 2013;51:361–70.
To T, Stanojevic S, Moores G, Gershon AS, Bateman ED, Cruz AA, et al. Global asthma prevalence in adults: findings from the cross-sectional world health survey. BMC Public Health. 2012;12:204.
Lin J, Wang W, Chen P, Zhou X, Wan H, Yin K, et al. Prevalence and risk factors of asthma in mainland China: The CARE study. Respir Med. 2018;137:48–54.
Huang K, Yang T, Xu J, Yang L, Zhao J, Zhang X, et al. China Pulmonary Health (CPH) Study Group. Prevalence, risk factors, and management of asthma in China: a national cross-sectional study. Lancet. 2019;394:407–18.
Ma YL, Zhang WB, Yu B, Chen YW, Mu S, Cui YL. Prevalence of allergic bronchopulmonary aspergillosis in Chinese patients with bronchial asthma. Zhonghua Jie He He Hu Xi Za Zhi. 2011;34:909–13.
Su N, Lin JT, Wang WY, Chen P, Zhou X, Wan HY, et al. A cross-section study of severe asthma in eight provinces of China. Zhonghua Nei Ke Za Zhi. 2016; 55:917–21.
Guan WJ, Gao YH, Xu G, Lin ZY, Tang Y, Li HM, et al. Aetiology of bronchiectasis in Guangzhou, southern China. Respirology. 2015; 20:739–48.
Severe asthma with fungal sensitization
Denning DW, Pleuvry A, Cole DC. Global burden of allergic bronchopulmonary aspergillosis with asthma and its complication chronic pulmonary aspergillosis in adults. Med mycol. 2013;51:361–70.
Zou H, Su L, Fang QH, Ma YM. Correlation between fungal sIgE and bronchial asthma severity. Exp Ther Med. 2013; 6:537–41.
Cryptococcal meningitis
Li Z, Liu Y, Cao H, Huang S, Long M. Epidemiology and clinical characteristics of cryptococcal meningitis in China (1981-2013): a review of the literature. Med Mycol: Open Access. 2017;03.
Chen J, Zhang R, Shen Y, Liu L, Qi T, Wang Z, et al. Serum cryptococcal antigen titre as a diagnostic tool and a predictor of mortality in HIV-infected patients with cryptococcal meningitis. HIV Med. 2018;20:69–73.

Invasive aspergillosis
Guo J, Zhou J, Zhang S, Zhang X, Li J, Sun Y, et al. A case–control study of risk factors for HIV-negative children with cryptococcal meningitis in Shi Jiazhuang, China. BMC Infect Dis. 2012;12:376.
Pneumocystis pneumonia
Chan CK, Alvarez Bognar F, Wong KH, Leung CC, Tan CK, Chan KC, et al. The epidemiology and clinical manifestations of human immunodeficiency virus-associated tuberculosis in Hong Kong. Hong Kong Med J. 2010;16:192–8.<br>Wang XL, Wei W, An CL. Retrospective study of pneumocystis pneumonia over half a century in mainland China. J Med Microbiol. 2011;60:631–38.<br>Guo F, Chen Y, Yang SL, Xia H, Li XW, Tong ZH. Pneumocystis pneumonia in HIV-Infected and immunocompromised non-HIV infected patients: a retrospective study of two centers in China. PLoS ONE. 2014; 9:e101943.<br>Hui M. *Pneumocystis carinii* pneumonia in Hong Kong: a 10 year retrospective study. J Med Microbiol. 2006;55:85–8.<br>Xiao J, Gao G, Li Y, Zhang W, Tian Y, Huang Y, et al. Spectrums of opportunistic infections and malignancies in HIV-infected patients in tertiary care hospital, China. PLoS ONE. 2013;8:e75915.<br>Chan CK, Alvarez Bognar F, Wong KH, Leung CC, Tam CM, Chan KC, et al. The epidemiology and clinical manifestations of human immunodeficiency virus-associated tuberculosis in Hong Kong. Hong Kong Med J. 2010;16:192–8.<br>Lowe DM, Rangaka MX, Gordon F, James CD, Miller RF. *Pneumocystis jirovecii* pneumonia in tropical and low and middle income countries: a systematic review and meta-regression. PLoS One. 2013;8:369969.
Histoplasmosis
Pan B, Chen M, Pan W, Liao W. Histoplasmosis: a new endemic fungal infection in China? Review and analysis of cases. Mycoses. 2012;56:212–21.
Talaromyces marneffei
Hu Y, Zhang J, Li X, Yang Y, Zhang Y, Ma J, et al. *Penicillium marneffei* infection: an emerging disease in mainland China. Mycopathologia. 2012;175:57–67.<br>Qi T, Zhang R, Shen Y, Liu L, Lowrie D, Song W, et al. Etiology and clinical features of 229 cases of bloodstream infection among chinese HIV/AIDS patients: a retrospective cross-sectional study. Eur J Clin Microbiol Infect Dis. 2016;35:1767–70.
Candidemia
Guo F, Yi Yang Y, Kang Y, Zang B, Cui M, Qin B, et al. Invasive candidiasis in intensive care units in China: a multicenter prospective observational study. J Antimicrob Chemother. 2013;68:1660–8.<br>Du B, An Y, Kang Y, Yu X, Zhao M, Ma X, et al. China Critical Care Clinical Trial Group. Characteristics of critically ill patients in ICUs in mainland China. Crit Care Med. 2013;41:84–92.<br>Tan BH, Chakrabarti A, Li RY, Patel AK, Watcharananan SP, Liu Z, et al. Incidence and species distribution of candidaemia in Asia: a laboratory-based surveillance study. Clin Microbiol Infect. 2015;21:946–53.<br>Montravers P, Mira J-P, Gangneux J-P, Leroy O, Lortholary O. A multicentre study of antifungal strategies and outcome of Candida spp. peritonitis in intensive-care units. Clin Microbiol Infect. 2011;17:1061–7.<br>Hu S, Tong R, Bo Y, Ming P, Yang H. Fungal peritonitis in peritoneal dialysis: 5-year review from a North China center. Infection. 2018;47:35–43.<br>Yang ZT, Wu L, Liu XY, Zhou M, Li J, Wu JY, et al. Epidemiology, species distribution and outcome of nosocomial Candida spp. bloodstream infection in Shanghai. BMC Infect Dis. 2014;14:241.

Invasive aspergillosis
Falagas ME, Roussos N, Vardakas KZ. Relative frequency of albicans and the various non-albicans Candida spp among candidemia isolates from inpatients in various parts of the world: a systematic review. Int J Infect Dis. 2010;14:e954–66.
Candida peritonitis (CAPD)
Wilkie M, Davies S. Insights on Peritoneal Dialysis in China. Perit Dial Int. 2018;38:S16–8.<br>Shouci H, Ren t, Yang B, Pei M, Hongtao Y. Fungal peritonitis dialysis: 5-year review from a North China center. Infection. 2019;47:35–43.
Esophageal candidiasis
Smith E, Orholm M. Trends and patterns of opportunistic diseases in Danish AIDS patients 1980–1990. Scand J Infect Dis. 1990;22:665–72.<br>Choi JH, Lee CG, Lim YJ, Kang HW, Lim CY, Choi JS. Prevalence and risk factors of esophageal candidiasis in healthy individuals: a single center experience in Korea. Yonsei Med J. 2013;54:160–5.
Oral candidiasis
Matee M, Scheutz F, Moshy J. Occurrence of oral lesions in relation to clinical and immunological status among HIV-infected adult Tanzanians. Oral Dis. 2008;6:106–11.<br>Denning DW. Minimizing fungal disease deaths will allow the UNAIDS target of reducing annual AIDS deaths below 500 000 by 2020 to be realized. Phil Trans Roy Soc B. 2016;371:20150468.
Recurrent vulvovaginal candidiasis
Denning DW, Kneale M, Sobel JD, Rautemaa-Richardson R. Global burden of recurrent vulvovaginal candidiasis. Lancet Infect Dis. 2018;18:e339–47.
Fungal keratitis
Song X, Xie L, Tan X, Wang Z, Yang Y, Yuan Y, et al. A multi-center, cross-sectional study on the burden of infectious keratitis in China. PLoS ONE. 2014;9:e113843.
Mucormycosis
Prakash H, Chakrabarti A. Global epidemiology of mucormycosis. J Fungi (Basel). 2019;5:26.
Onychomycosis
Sigurgeirsson B, Baran R. The prevalence of onychomycosis in the global population: a literature study. J Eur Acad Dermatol Venereol. 2014;28:1480–91.
*Reports published in English during January 1950–October 2019 were searched.

Appendix Table 4. Studies contributing to epidemiology map of talaromycosis in China*

Province	No. cases
Zhejiang Province	
Si Z, Qiao J. *Talaromyces marneffei* infection. N Engl J Med. 2017;377;2580.	1
Yu X, Cai X, Xu X, Zhang L, Huang X, Wang L, et al. Fungemia caused by *penicillium marneffei* in an immunocompetent patient with COPD: a unique case report. Medicine (Baltimore). 2018; 97:e9658.	1
Yu X, Miao K, Zhou C, Cai Y, Huang X, Chen Y, et al. *T. Marneffei* infection complications in an HIV-negative patient with pre-existing pulmonary sarcoidosis: a rare case report. BMC Infect Dis. 2018;18;390.	1
Wang P, Chen Y, Xu H, Ding L, WuZ, Xu Z. Acute disseminated *Talaromyces marneffei* in an immunocompetent patient. Mycopathologia. 2017;182; 751–4.	1
Huang SS, Zhang SN, Ye JR, Su SS, Lin PC, Li YP, et al. Diagnostic performance of pathology, culture and ROSE of lung biopsy for suspected pulmonary infectious diseases. 2019;99:3340–4.	3
Ge Y, Xu Z, Hu Y, Huang M. Successful voriconazole treatment of *Talaromyces marneffei* infection in an HIV-negative patient with osteolytic lesions. J Infect Chemother. 2019;25:204–7.	1
Su SS, Zhang SN, Ye JR, Xu LN, Lin PC, Xu HY, et al. Disseminated *Talaromyces marneffe*i and *Mycobacterium avium* infection accompanied Sweet's syndrome in a patient with anti-interferon-γ. Infect Drug Resist. 2019;12:3189–95.	1
Cui Y, Jin C, Li X, Wu N. *Penicillium marneffei* infection presenting as vulv-ulcer in an HIV-infected woman. Eur J Med Res. 2011;16:426–6.	1
Xia XJ, Shen H, Xu AE. Cutaneous *Penicillium marneffei* infection in a patient with idiopathic CD4(+) lymphocytopenia. J Dermatol. 2015;42:812–4.	1
Hong Kong	
Wong SCY, Sridhar S, Ngan AHY, Chen JHK, Poon RWS, Lau SKP, et al. Fatal *Talaromyces marneffei* infection in a patient with autoimmune hepatitis. Mycopathologia. 2018;183:615–8.	1
Hung HG, Lok KH. Intestinal *Penicillium marneffei*: an unusual cause of chronic diarrhea in an AIDS patient. J Dig Dis. 2010;11:189–91.	1
Chan YH, Wong KM, Lee KC, Kwok PC, Chak WL, Choi KS, et al. Pneumonia and mesenteric lymphadenopathy caused by disseminated *Penicillium marneffei* infection in a cadaveric renal transplant recipient. Transpl Infect Dis. 2004;6:28–32.	1
Lee PP, Lao-Araya M, Yang J, Chan KW, Ma H, Pei LC, et al. Application of flow cytometry in the diagnostics pipeline of primary immunodeficiencies underlying disseminated *Talaromyces marneffei* infection in HIV-negative children. Front Immunol. 2019;10:2189.	8
Wong KH, Lee SS. Comparing the first and second hundred AIDS cases in Hong Kong. Singapore Med J. 1998;39:236–40.	19
Wu TC, Chan JW, Ng CK, Tsang DN, Lee MP, Li PC. Clinical presentations and outcomes of *Penicillium marneffei* infections: a series from 1994 to 2004. Hong Kong Med J. 2008;14:103–9.	47
Ma BH, Ng CS, Lam R, Wan S, Wan IY, Lee TW, et al. Recurrent hemoptysis with *Penicillium marneffei* and *Stenotrophomonas maltophilia* in Job's syndrome. Can Respir J. 2009;16:e50–2.	1

Province	No. cases
Wong SS, Woo PC, Yuen KY. *Candida tropicalis* and *Penicillium marneffei* mixed fungaemia in a patient with Waldenström's macroglobulinaemia. Eur J Clin Mircobiol Infect Dis. 2001;20:132–5.	1
Guangdong Province	
Lu S, Li X, Calderone R, Zhang J, Ma J, Cai W, et al. Whole blood nested PCR and real-time PCR amplification of *Talaromyces marneffei* specific DNA for diagnosis. Med Mycol. 2016;54:162–8.	23
Lei M, Yu U, Zhang N, Deng J. An HIV-negative infant with systemic *Talaromyces marneffei* infection. Int J Infect Dis. 2018;77:3–4.	1
Chi XH, Xue YM, Wang QS, Li GP, Zhou HS, Qi YS. Diagnosis and treatment of diffusible *Penicillium marneffei* in human immunodeficiency virus-negative patients: a challenge for the physician. Indian J Med Microbiol. 2017;35:617–9.	1
Li HR, Xu NL, Lin M, Hu XL, Chen JH, Chen YS, et al. Diffuse interstitial and multiple cavitary lung lesions due to *Talaromyces marneffei* infection in a non-HIV patient. New Microbes New Infect. 2015;8:14–6.	1
Li LH, Hu FY, Chen WS, Cai WP, Song WN, Kuang YL, et al. Genetic diversity analysis of *Penicillium marneffei* isolated from AIDS patients in Guangdong, China using randomly amplified polymorphic DNA. Chin Med J (Engl). 2012;125:823–7.	163
Zhou F, Bi X, Zou X, Xu Z, Zhang T. Retrospective analysis of 15 cases of *Penicilliosis marneffei* in a southern China hospital. Mycopathologia. 2014;177:271–9.	15
Zhang J, Huang X, Zhang X, Zhu Y, Liao K, Ma J. Coinfection of disseminated *Talaromyces marneffei* and *Mycobacteria kansasii* in a patient with papillary thyroid cancer: a case report. Medicine (Baltimore). 2017;96:e9072.	1
Wang YF, Xu HF, Han ZG, Zeng L, Liang CY, Chen YJ. Serological surveillance for *Penicillium marneffei* infection in HIV-infected patients during 2004–2011 in Guangzhou, China. Clin Microbiol Infect. 2015;21:484–9.	761
Lau SKP, Xing F, Tsang CC, Tang JYM, Tan YP, Ye H, et al. Clinical characteristics, rapid identification, molecular epidemiology and antifungal susceptibilities of *Talaromyces marneffei* infections in Shenzhen, China. Mycoses. 2019;62:450–7.	7
Fan H, Huang L, Yang D, Lin Y, Lu G, Xie Y, et al. Pediatric hyperimmunoglobulin E syndrome: a case series of 4 children in China. Medicine (Baltimore). 2018;97:e0215.	1
Luo DQ, Chen MC, Liu JH, Li Z, Li HT. Disseminated *Penicillium marneffei* infection in an SLE patient: a case report and literature review. Mycopathologia. 2011;171:191–6.	1
Peng J, Chen Z, Cai R, Huang X, Lin L, Liang W, et al. Recovery from *Talaromyces marneffei* involving the kidney in a renal transplant recipient: a case report and literature review. Transpl Infect Dis. 2017;19.	1
Liyan X, Changming L, Xianyi Z, Luxia W, Suisheng X. Fifteen cases of penicilliosis in Guangdong, China. Mycopathologia. 2004;158:151–5.	15
Xian J, Hunag X, Li Q, Peng X, Peng X. Dermatoscopy for the rapid diagnosis of *Talaromyces marneffei* infection: a case report. BMC Infect Dis. 2019;19:707.	1
Lao M, Zhan Z, Su F, Li H, Yang Z, Chen H, et al. Invasive mycoses in patients with connective tissue disease from Southern China: clinical features and associated factors. Arthritis Res Ther. 2019; 21:71.	2
Lu PX, Zhu WK, Liu Y, Chen XC, Zhan NY, Liu JQ, et al. Acquired immunodeficiency syndrome associated with disseminated *Penicillium marneffei* infection: report of 8 cases. Chin Med J (Engl). 2005; 118:1395–9.	8

Province	No. cases
Ye F, Luo Q, Zhou Y, Xie J, Zeng Q, Chen G, et al. Disseminated *Penicilliosis marneffei* in immunocompetent patients: a report of two cases. Indian J Med Microbiol. 2015;33:161–5.	2
Du Q, Tong Ck. *Talaromyces (Penicillium) marneffei* infection. IDCases. 2018;13:e00428.	1
Li Q, Wang C, Zeng K, Peng X,Wang F. AIDS-associated disseminated *talaromycosis (penicilliosis) marneffei.*J Dtsch Dermatol Ges. 2018;16:1256–9.	1
Beijing	
Liu X, Wu H, Huang X. Disseminated *Penicillium marneffei* infection with IRIS. IDCases. 2015; 2:92–3.	1
Li H, Sang J, Li R, Liu Y, Zhang J. Disseminated *Penicillium Marneffei* infection with verrucoid lesions in an AIDS patient in Beijing, a non-endemic region. Eur J Dermatol. 2010;20:378–80.	1
Lu ZH, Liu HR, Xie XL, Wang AX, Liu TH. Infection of *Penicillium marneffe.* Zhonghua Bing Li Xue Za Zhi. 2004;33:53640.	1
Xiao J, Gao G, Li Y, Zhang W, Tian Y, Huang Y, et al. Spectrums of opportunistic infections and malignancies in HIV-infected patients in tertiary care hospital, China. PLoS One. 2013;8:e75971.	11
Zhao DW, Zhang T, Ma DQ, Wang W, Yuan CW, Duan Y. Disseminated *Penicillium marneffei* infection in acquired immunodeficiency syndrome: a case report. Chin Med J (Engl), 2005;118:1054–6.	1
Qu H, Song Y, Li R, Yu J. Image gallery: an unusual cutaneous presentation of disseminated *penicilliosis marneffei* in an immunocompetent patient. Br J Dermatol. 2017;177:e67.	1
Jiang X, Zhou D. Diagnosis of *Penicillium marneffei* infection from a blood film. Br J Haematol. 2015;171:670.	1
Guangxi Province	
Liu GN, Huang JS, Zhong NX, Zhang JQ, Zou ZX, Yang ML, et al. *Penicillium marneffei* infection within an osteolytic lesion in an HIV-negative patient. Int J Infect Dis. 2014;23:1–3.	1
Li X, ZhengY, Wu F, Mo D, Liang G, Yan R, et al. Evaluation of quantitative real-time PCR and Platelia galactomannan assays for the diagnosis of disseminated *Talaromyces marneffei* infection. Med Mycol. 2019 May 27. Pii:myz052.	36
Qiu Y, Zeng W, Zhang H, Zhang X, Tang S, Zhang J. Comparison of pleural effusion features and biomarkers between talaromycosis and tuberculosis in non-human immunodeficiency virus-infected patients. BMC Infect Dis. 2019;19:745.	19
Deng Z, Ribas JL, Gibson DW, Connor DH. Infections caused by *Penicillium marneffei* in China and Southeast Asia: review of eighteen published cases and report of four more Chinese cases. Rev Infect Dis. 1988;10:640–52.	4
Deng Z, Liu x. Disseminated *Penicilliosis marneffei* in a patient with acquired immunodeficiency syndrome: a first case report from China. Chin Med J (Engl). 2000;113:1049–50.	1
Jiang J, Meng S, Huang S, Ruan Y, Lu X, Li JZ, et al. Effects of *Talaromyces marneffei* infection on mortality of HIV/AIDS patients in southern China: a retrospective cohort study. Clin Microbiol Infect. 2019;25:233–41.	1093
Ouyang Y, Cai S, Liang H, Cao C. Administration of voriconazole in disseminated *Talaromyces (Penicillium) Marneffei* infection: a retrospective study. Mycopathologia. 2017;182:569–75.	112
Shi N, Kong J, Wang K, Cao C. Coinfection with *Talaromyces marneffei* and other pathogens associated with acquired immunodeficiency. JAMA Dermatol. 2019 Jul 24.	1

Province	No. cases
Xu H, Liu D, He X, Zheng D, Deng Y. Sweet's syndrome associated with *Talaromyces marneffei* and *Mycobacterium abscessus* infection due to anti-interferon-gamma autoantibodies. Indian J Dermatol. 2018;63:428–30.	1
Han J, Lun WH, Meng ZH, Huang K, Mao Y, Zhu W, et al. Mucocutaneous manifestations of HIV-infected patients in the era of HAART in Guangxi Zhuang Autonomous Region, China. J Eur Acad Dermatol Venereol. 2013;27:376–82.	40
Guo J, Li BK, Li TM, Wei FL, Fu YJ, Zheng YQ, et al. Characteristics and prognosis of *Talaromyces marneffei* infection in non-HIV-infected children in southern China. Mycopathologia. 2019 Aug 31.	11
Qiu Y, Zhang J, Liu G, Zhong X, Deng J, He Z, et al. Retrospective analysis of 14 cases of disseminated *Penicillium marneffei* infection with osteolytic lesions. BMC Infect Dis. 2015;15:47.	14
Lin F, Qiu Y, Zeng W, Liang Y, Zhang J. *Talaromyces marneffei* infection in a lung cancer patient: a rare case report. BMC Infect Dis. 2019;19:336.	1
Zeng W, Qiu Y, Lu D, Zhang J, Zhong X, Liu G. A retrospective analysis of 7 human immunodeficiency virus-negative infants infected by *Penicillium marneffei*. Medicine (Baltimore). 2015;94:e1439.	7
Qiu Y, Liao H, Zhang J, Zhong X, Tan C, Lu D. Differences in clinical characteristics and prognosis of penicilliosis among HIV-negative patients with or without underlying disease in southern China: a retrospective study. BMC Infect Dis. 2015;15:525.	109
Qiu Y, Pan M, Zhang J, Zhong X, Li Y, Zhang H, et al. Two unusual cases of human immunodeficiency virus-negative patients with *Talaromyces marneffei* infection. Am J Trop med Hyg. 2016;95:426–30.	2
Qin L, Zhao L, Tan C, Chen XU, Yang Z, Mo W. A novel method of combining periodic acid Schiff staining with Wright-Giemsa staining to identify the pathogens *Penicillium marneffei*, *Histoplasma capsulatum*, *Mucor* and *Leishmania donovani* in bone marrow smears. Exp Ther Med. 2015;9:1950–4.	25
Li JS, Pan LQ, Wu SX, Su SX, Su SB, Shan LY. Disseminated *penicilliosis marneffei* in China. Report of three cases. Chin Med J (Engl). 1991;104:247–51.	3
Zeng W, Qiu Y, Tang S, Zhang J, Pan M, Zhong X. Characterization of anti-interferon-γ antibodies in HIV-negative patients infected with disseminated *Talaromyces marneffei* and cryptococcosis. Open Forum Infect Dis. 2019;6:ofz208.	20
Qiu Y, Zhang JQ, Pan ML, Zeng W, Tang SD, Tan CM. Determinants of prognosis in *Talaromyces marneffei* infections with respiratory system lesions. Chin Med J (Engl). 2019;132:1909–18.	126
Qiu Y, Zhang J. Behcet's disease with pulmonary artery aneurysm and *Talaromyces marneffei*. Int J Infect Dis. 2017;54:34–5.	1
Qiu Y, Lu D, Zhang J, Zhong X, Liu G, Li B. Treatment of disseminated *Talaromyces marneffei* with tracheal infection: two case reports. Mycopathologia. 2015;180:245–9.	2
Mo W, Deng Z, Li S. Clinical blood routine and bone marrow smear manifestations of disseminated *Penicilliosis marneffei*. Chin Med J (Engl). 2002;115:1892–4.	13
Li Y, Lin Z, Shi X, Mo L, Li W, Mo W, et al. Retrospective analysis of 15 cases of *Penicillium marneffei* infection in HIV-positive and HIV-negative patients. Microb Pathog. 2017;105:321–5.	15
Wei XG. Report of the first case of *penicilliosis marneffei* in China. Zhonghua Yi Xue Za Zhi. 1985; 65:533–4.	1

Province	No. cases
Qiu Y, Tang Y, Zhang J, Yi X, Zhong X, Liu G, Xu H, et al. A retrospective analysis of seven patients with acquired immunodeficiency syndrome and pharyngeal and/or laryngeal *Talaromyces marneffei* infection. Clin Otolaryngol. 2017;42:1061–6.	7
Nong S, Liang J. Bone marrow *Penicillium marneffei* infection in acquired immunodeficiency syndrome patients: report of 35 cases. Trop Biomed. 2013;30:89–91.	35
Shanghai	
Qi T, Zhang R, Shen Y, Liu L, Lowrie D, Song W, et al. Etiology and clinical features of 229 cases of bloodstream infection among Chinese HIV/AIDS patients: a retrospective cross-sectional study. Eur J Clin Microbiol Infect Dis. 2016;35:1767–70.	43
Chen J, Zhang R, Shen Y, Liu L, Qi T, Wang Z, et al. Clinical characteristics and prognosis of penicilliosis among human immunodeficiency virus-infected patients in eastern China. Am J Trop Med Hyg. 2017;96:1350–4.	48
Chen M, Houbraken J, Pan W, Zhang C, Peng H, Wu L, et al. Pulmonary fungus ball caused by *Penicillium capsulatum* in a patient with type 2 diabetes: a case report. BMC Infect Dis. 2013;13:496.	2
Zhang HC, Zhang QR, Ai JW, Cui P, Wu HL, Zhang WH, et al. The role of bone marrow metagenomics next-generation sequencing to differential diagnosis among visceral leishmaniasis, histoplasmosis, and *talaromycosis marneffei*. Int J lab Hematol. 2019 Oct 8.	1
Zhu YM, Ai JW, Xu B, Cui P, Cheng Q, Wu H, et al. Rapid and precise diagnosis of disseminated *T. marneffei* infection assisted by high-throughput sequencing of multifarious specimens in a HIV-negative patient: a case report. BMC Infect Dis. 2018;18:379.	1
Zhu LP, Yang FF, Weng XH, Huang YX, Chen S, Shi GF, et al. Hepatic safety of itraconazole intravenous solution in treatment of invasive fungal infection. Zhonghua Yi Xue Za Zhi. 2006; 86:2028–32.	1
Shen YZ, Wang ZY, Lu HZ. *Penicillium marneffei* chylous ascites in acquired immune deficiency syndrome: a case report. World J Gastronterol. 2012;18:5312–4.	1
Fujian Province	
Li HR, Cai SX, Chen YS, Yu ME, Xu NL, Xie BS, et al. Comparison of *Talaromyces marneffei* infection in human immunodeficiency virus-positive and human immunodeficiency virus-negative patients from Fujian, China. Chin Med J (Engl). 2016;129:1059–65.	26
Lai JL, Chen YH, Liu YM, Yuan JJ, Lin J, Huang AQ, et al. Prevalence and risk factors of anaemia in hospitalised HIV-infected patients in southeast China: a retrospective study. Epidemiol Infect. 2019; 147:e81.	61
Chen Y, Huang A, Ao W, Wang Z, Yuan J, Song Q, et al. Proteomic analysis of serum proteins from HIV/AIDS patients with *Talaromyces marneffei* infection by TMT labeling-based quantitative proteomics. Clin Proteomics. 2018;15:40.	18
Wang YG, Cheng JM, Ding HB, Lin X, Chen GH, Zhou M, et al. Study on the clinical features and prognosis of *Penicilliosis marneffei* without human immunodeficiency virus infection. Mycopathologia. 2018;183:551–8.	6
Hubei Province	
Han P, Yan W, Luo Y, Tu W, He JY, Liu JM, et al. Chronic bronchitis with fungal infection presenting with marked elevation of serum carbohydrate antigen 19-9: a case report. Int J Clin Exp Pathol. 2014;7:6307–12.	1

Province	No. cases
Liu XY, Chen KL, You Y, Chen WX, Zou P. The differential features of bone marrow morphology of several rare infectious disorders. Zhoughua Nei Ke Za Zhi. 2005;44:902–5.	1
Xiang Y, Guo W, Liang K. An unusual appearing skin lesion from *Penicillium marneffei* infection in an AIDS patient in central China. Am J Trop Med Hyg. 2015;93:3.	1
Sichuan Province	
Pang W, Shang P, Li Q, Xu J, Bi L, Zhong J, et al. Prevalence of opportunistic infections and causes of death among hospitalized HIV-infected patients in Sichuan, China. Tohoku J Exp Med. 2018; 244:231–42.	3
Liao X, Ran Y, Chen H, Meng W, Xiang B, Kang M, et al. Disseminated *Penicillium marneffei* infection associated with AIDS, report of a case. Zhonghua Yi Xue Za Zhi. 2002;82:326–9.	1
Xu X, Ran X, Pradhan S, Lei S, Ran Y. Dermoscopic manifestations of *Talaromyces (Penicillium) marneffei* infection in an AIDS patient. Indian J Dermatol Venereol Leprol. 2019;85:348.	1
Zhiyong Z, Mei K, Yanbin L. Disseminated *Penicillium marneffei* infection with fungemia and endobronchial disease in an AIDS patient in China. Med Princ Pract. 2006;15:235–7.	1
Hunan Province	
Zhang Z, Tao F, Li Y, Xiao Y, Zhang Z, Liu J. Disseminated *Penicillium marneffei* infection recurrence in a non-acquired immune deficiency syndrome patient: a case report. Mol Clin Oncol. 2016;4:829–31.	1
Liu D, Zhong LL, Li Y, Chen M. Recurrent fever, hepatosplenomegaly and eosinophilia in a boy. Zhongguo Dang Dai Er Ke Za Zhi. 2016;18:1145–9.	1
Yunnan Province	
Li YY, Saeed U, Wei SS, Wang L, Kuang YQ. Both coinfections of *Penicillium marneffei* and *Cryptococcus neoformans* in AIDS patient: a report of rare case. AIDS. 2017;31:2171–2.	1
Taiwan	
Yen YF, Chen M, Jen I, Lan YC, Chuang PH, Liu YL, et al. Association of HIV and opportunistic infections with incident stroke: a nationwide population-based cohort study in Taiwan. J Acquir Immune Defic Syndr. 2017;74:117–25.	126
Cheng NC, Wong WW, Fung CP, Liu CY. Unusual pulmonary manifestations of disseminated *Penicillium marneffei* infection in three AIDS patients. Med Mycol. 1998;36:429–32.	3
Chang cc, Liao ST, Huang WS, Liu JD, Shih LS. Disseminated *Penicillium marneffei* infection in a patient with acquired immunodeficiency syndrome. J Formos Med Assoc. 1995;94:527–35.	1

*We searched the PubMed database for articles published in China during January 1, 1950–October 7, 2019, using ‘‘*penicillium marneffeii*,” ‘‘talaromycosis,’’ or ‘‘penicilliosis’’ as the search strings for talaromycosis.

Appendix Table 5. Studies contributing to epidemiology map of histoplasmosis in China*

Province	No. cases
Hunan Province	
Zhang Y, Su X, Li Y, He R, Hu C, Pan P. Clinical comparative analysis for pulmonary histoplasmosis and progressive disseminated histoplasmosis. Zhong Nan Da Xue Xue Bao Yi Xue Ban. 2016;41:1345–51.	12
Zhou L, Fan S, Liang Q, Peng Y, Zong D, Ouyang R. Clinical characteristics of histoplamosis in 8 patients: case report and literature review. Zhong Nan Da Xue Xue Bao Yi Xue Ban. 2016;41:644–52.	8
Zhao B, Xia X, Yin J, Zhang X, Wu E, Shi Y, et al. ,Epidemiological investigation of *Histoplasma capsulatum* infection in China. Chin Med J (Engl). 2001;114:743–6.	47
Zhu C, Wang G, Chen Q, He B, Wang L. Pulmonary histoplasmosis in a immunocompetent patient: a case report and literature review. Ex Ther Med. 2016;12:3256–60.	1
Clinical features and endemic trend of histoplasmosis in China: a retrospective analysis and literature review. Clin Respir J. 2019; Dec 07.	34
Sichuan Province	
Zhu LL, Wang J, Wang ZJ, Wang Yp, Yang JL. Intestinal histoplasmosis in immunocompetent adults. World J Gastroenterol. 2016;22:4027–33.	1
Yang B, Lu L, Li D, Liu L, Huang L, Chen L, et al. Colonic involvement in disseminated histoplasmosis of an immunocompetent adult: case report and literature review. BMC Infect Dis, 2013;13:143.	1
Wen FQ, Sun YD, Watanabe K, Yoshida M, Wu JN, Baum GL. Prevalence of histoplasmin sensitivity in healthy adults and tuberculosis patients in southwest China. J Med Vet Mycol. 1996;34:1714.	67
Xiong XF, Fan LL, Kang M, Wei J, Cheng DY. Disseminated histoplasmosis: a rare clinical phenotype with difficult diagnosis. Respirol Case Rep. 2017; 5:e00220.	1
Beijing	
Gong P, Cao Z, Mu X, Dong X, Wang K, Feng R, et al. The clinical-radiologic-pathologic features of imported pulmonary histoplasmosis. Zhonghua Jie He He Hu Xi Za Zhi, 2015;38:23–8.	3
Zhao CS, Zhao SY, Liu G, Xi-Wei X. Risk factors of invasive fungal infections in patients admitted to non-hematological oncology department and pediatric intensive care unit. Zhonghua Er Ke Za Zhi. 2013;51:589–601.	1
Zhejiang Province	
Liu B, Qu L, Zhu J, Yang Z, Yan S. Histoplasmosis mimicking metastatic spinal tumour. J Int med Res. 2017;45:1440–6.	1
Shen G, Chai Y, Zhang GF, Wei HQ, Yue L. Diagnosis and treatment of pulmonary histoplasmoma: report of 3 cases. Zhonghua Yi Xue Za Zhi. 2007;87:760–2.	3
Ye C, Zhang G, Wang J, Chai Y. Histoplasmosis presenting with solitary pulmonary nodule: two cases mimicking pulmonary metastases. Niger J Clin Pract. 2015;18:304–6.	2
Zhang X, Jin J, Cai C, Zheng R, Wang Y, Xu Y. Amphotericin B liposome-induced acrocyanosis and elevated serum creatinine. Indian J Pharmacol. 2016;48:321–3.	1
Huang L, Wu Y, Miao X. Localized *Histoplasma capsulatum* osteomyelitis of the fibula in an immunocompetent teenage boy: a case report. BMC Infect Dis. 2013;13:132.	1

Province	No. cases
Shanghai	
Wang Y, Pan B, Wu J, Bi X, Liao W, Pan W, et al. Detection and phylogenetic characterization of a case of *Histoplasma capsulatum* infection in mainland China. Am J Trop med Hyg. 2014;80:1180–3.	1
Zhang HC, Zhang QR, Ai JW, Cui P, Wu HL, Zhang WH, et al. The role of bone marrow metagenomics next-generation sequencing to differential diagnosis among visceral leishmaniasis, histoplasmosis, and *talaromycosis marneffei*. Int J lab Hematol. 2019 Oct 8.	1
Guangxi Province	
Qin L, Zhao L, Tan C, Chen XU, Yang Z, Mo W. A novel method of combining periodic acid Schiff staining with Wright-Giemsa staining to identify the pathogens *Penicillium marneffei*, *Histoplasma capsulatum*, *Mucor* and *Leishmania donovani* in bone marrow smears. Exp Ther Med. 2015;9:1950–4.	10
Li ZS. Histoplasmosis in south Guangxi (report of 5 cases). Zhonghua Yi Xue Za Zhi. 1982;62:267–9.	5
Cao C, Bulmer G, Li J, Liang L, Lin Y, Xu Y, et al. Indigenous case of disseminated histoplasmosis from the *Penicillium marneffei* endemic area of China. Mycopathologia. 2010;170:4750.	1
Jiangsu Province	
Zhao B, Xia X, Yin J, Zhang X, Wu E, Shi Y. Epidemiological investigation of *Histoplasma capsulatum* infection in China. Chin Med J (Engl). 2001;114:743–6.	80
Lü PH, Zhao BL, Shi Y, Wen YT. The diagnostic value of detecting plasma 1,3-beta-D-glucan for invasive fungal infections. Zhonghua Jie He He Hu Xi Za Zhi, 2007;30:31–4.	1
Zhao B, Yin J, Xia X. Investigation on the epidemiology of *Histoplasma capsulatum* infection in Nanjing district. Zhonghua Liu Xing Bing Xue Za Zhi. 1998;19:215–7.	49
Xinjiang Province	
Zhao B, Xia X, Yin J, Zhang X, Wu E, Shi Y, et al. Epidemiological investigation of *Histoplasma capsulatum* infection in China. Chin Med J (Engl). 2001;114:743–6.	11
Taiwan	
Liu JW, Huang TC, Lu YC, Liu HT, Li CC, Wu JJ, et al. Acute disseminated histoplasmosis complicated with hypercalcaemia. J Infect. 1999;39:88–90.	1
Tseng TC, Liaw SJ, Hsiao CH, Wang CY, Lee LN, Huang TS, et al. Molecular evidence of recurrent histoplasmosis with 9-year latency in a patient with Addison's disease. J Clin Microbiol. 2005; 43:4911–3.	1
Chang YG, Chen PJ, Hung CC, Chen MY, Lai mY, Chen DS. Opportunistic hepatic infections in AIDS patients with fever of unknown origin. J Formos Med Assoc. 1999;98:5–10.	1
Lai CH, Huang CK, Chin C, Yang YT, Lin HF, Lin HH. Indigenous case of disseminated histoplasmosis, Taiwan. Emerg Infect Dis. 2007;13:127–9.	1
Chang YT, Huang SC, Hu SY, Tsan YT, Wang LM, Wang RC. Disseminated histoplasmosis presenting as haemolytic anaemia. Postgrad Med J. 2010;86:443–4.	1
Chongqing Province	
Ge L, Zhou C, Song Z, Zhang Y, Wang L, Zhng B, et al. Primary localized histoplasmosis with lesions restricted to the mouth in a Chinese HIV-negative patient. Int j Infect Dis. 2010;14 Suppl 3:e325–8.	1
Guangdong Province	

Province	No. cases
Ai XB, Wang ZJ, Dong QC, Lin X, Chen YP, Gong FY. Ileum histoplasmosis mimicking intestinal tuberculosis and Crohn's disease. Case Rep Gastroenterol. 2018;12:63–8.	1
Huang LF, Tang XP, Cai WP, Chen XJ, Lei CJ, Li LH, et al. An analysis of opportunistic infection in 762 inpatients with human immunodeficiency virus infection in Guangdong areas. Zhonghua Nei Ke Za Zhi, 2010;49:653–6.	1
Meng Y, Cai S, Li X. Pathologically confirmed histoplasmosis: analysis of 14 cases. Nan Fang Yi Ke Da Xue Xue Bao. 2013;33;296–8.	14
Dang Y, Jiang L, Zhang J, Pan B, Zhu G, Zhu F, et al. Disseminated histoplasmosis in an immunocompetent individual diagnosed with gastrointestinal endoscopy: a case report. BMC Infect Dis. 2019;19:992.	1
Hubei Province	
Han P, Yan W, Luo Y, Tu W, He JY, Liu JM, et al. Chronic bronchitis with fungal infection presenting with marked elevation of serum carbohydrate antigen 19-9: a case report. Int J Clin Exp Pathol. 2014; 7:6307–12.	1
Liu XY, Chen KL, You Y, Chen WX, Zou P. The differential features of bone marrow morphology of several rare infectious disorders. Zhonghua Nei Ke Za Zhi. 2005;44:902–5.	7
Li X, Li J, Feng GQ, Gui XE, Zeng XC. A primary investigation on disseminated histoplasmosis in Hubei. Zhonghua Liu Xing Bing XUe Za Zhi. 2003;24:708–10.	1
Hong Kong	
Wong KF, Cheng NH. Fever and productive cough in a patient with AIDS. Clin Infect Dis. 2011; 52:646–7.	1
Tsui WM, Ma KF, Tsang DN. Disseminated *Penicillium marneffei* infection in HIV-infected subject. Histopathology. 1992;20:287–93.	4

* We searched the PubMed database for articles published in China during January 1, 1950–October 7, 2019, using “histoplasmosis” or “*Histoplasma*” for histoplasmosis.
